# Mass spectrometry and molecular modeling studies on the inclusion complexes between alendronate and β-cyclodextrin

**DOI:** 10.1007/s10847-013-0315-0

**Published:** 2013-04-17

**Authors:** Joanna Biernacka, Katarzyna Betlejewska-Kielak, Janina Witowska-Jarosz, Ewa Kłosińska-Szmurło, Aleksander P. Mazurek

**Affiliations:** 1 Department of Drug Chemistry, Medical University of Warsaw, 1 Banacha Street, 02-097 Warsaw, Poland; 2 National Medicines Institute, 30/34 Chełmska Street, 00-725 Warsaw, Poland

**Keywords:** β-Cyclodextrin, Alendronate sodium, Inclusion complex, Mass spectrometry, Electrospray ionization, Molecular modeling

## Abstract

Complexation of alendronate sodium (AlnNa) with β-cyclodextrin (β-CD) was studied by means of ESI-mass spectrometry. The experimental results show that stable 1:1 inclusion complexes between selected bisphosphonates and β-CD were formed. In addition, complexes with different stoichiometry were observed. DFT/B3LYP calculations were performed to elucidate the different inclusion behavior between alendronate and β-CD. Molecular modeling showed that the inclusion complex of Aln-β-CD where the two phosphonate groups bound to the central carbon atom of bisphosphonate were inserted into the cavity of β-CD from its “top” side was thermodynamically more favorable than when they were inserted from its “bottom” side; the complexation energy was −74.05 versus −60.85 kcal/mol. The calculations indicated that the formation of conventional hydrogen bonds was the main factor for non-covalent β-CD:Aln complex formation and stabilization in the gas phase.

## Introduction

**Fig. 1:**
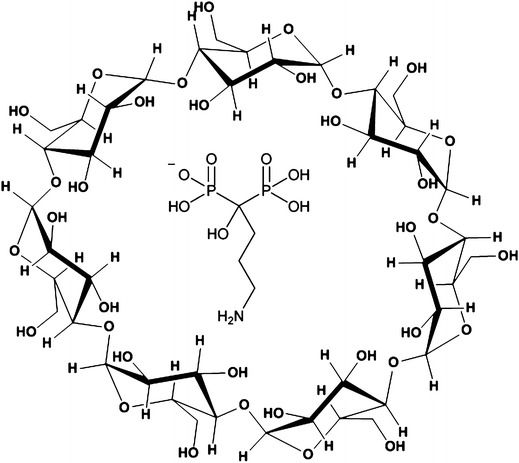
An unoptimized structure of the alendronate and β-CD complex

Alendronate sodium (AlnNa, Fig. 1) is a drug from the bisphosphonate group, which is commonly used to treat diseases that result from increased bone resorption. It is used in postmenopausal, senile and steroid-induced osteoporosis as well as for treatment of the Paget’s bone disease and bone metastases [1, 2]. Bisphosphonates are characterized by two phosphonate groups bound to the central carbon atom. They are analogues of pyrophosphate in which the central oxygen atom is replaced by a carbon atom, resulting in a P–C–P group. Bisphosphonates are characterized by a very low bioavailability [3]. After oral intake, they are absorbed through the gastrointestinal tract at a low rate. Furthermore, orally administered bisphosphonates may cause irritation and damage to the esophageal mucosa [4, 5]. One possibility to improve the bioavailability of the drug substance and to reduce its harmful effects is to produce complexes with macrocyclic oligosaccharides, for example β-cyclodextrin.

β-Cyclodextrin (β-CD, Fig. 1) is a macrocyclic oligosaccharide consisting of seven D-(+)glucopyranose units linked by α-1,4-glycosidic bonds. β-CD has an internal cavity with a truncated cone shape. It is about 8 Å deep and 6.0–6.5 Å wide. The 14 secondary hydroxyls on the C-2 and C-3 carbon atoms of the glucopyranose units are situated at the wider rim and the seven primary hydroxyls on the C-6 carbons are situated on the narrower rim of the cone. Most hydrogen atoms are directed to the middle part of the cone, resulting in a rather hydrophobic cavity along with edges of a more hydrophilic character [6, 7].

The complexation of drugs with cyclodextrins has been widely used in the pharmaceutical industry. The first pharmaceutical product in the world, prostaglandin E2/β-CD (Prostarmon E™ sublingual tablets), was marketed in Japan in 1976 by Ono Pharmaceutical Co. Twelve years later, the first European cyclodextrin-containing pharmaceutical product, piroxicam/β-CD (Brexin^®^ tablets), was marketed and in 1997, the first US-approved product, itraconazole/2-hydroxypropyl-β-CD oral solution (Sporanox^®^) was introduced [8, 9]. Nowadays 30–40 different drugs are marketed worldwide in different types of cyclodextrin complex formulations, including different types of tablets (i.e., conventional, chewing and sublingual tablets), oral capsules, parenteral solutions, suppositories, nasal sprays, eye drop solutions and dermal products [7]. The inclusion of drug molecules into CDs leads to important modifications to the physical, chemical and biological properties of the molecules. Complexation can potentially enhance the solubility of insoluble drugs, improve the efficacy, chemical stability and bioavailability of poorly soluble drugs, reduce their toxicity by making the drugs effective at lower doses, and control their rate of release [10, 11]. It can also moderate drug odors and flavors [12].

The ability of β-CD to form inclusion complexes with drug substances is mainly due to hydrophobic interactions, van der Waals and electrostatic interactions, as well as the formation of hydrogen bonds [13, 14]. Due to the lack of covalent bonds in these complexes, CDs are treated as excipients, and the resulting complexes are not considered as new active substances [15]. β-CD is mentioned in the US Pharmacopoeia (USP/NF), European Pharmacopoeia (Ph. Eur.) and also in the Japanese Pharmaceutical Codex (JPC). In 1995, the Joint FAO/WHO Expert Committee on Food Additives (JECFA) announced the ADI (Acceptable Daily Intake) value for β-CD at 0–5 mg/bw kg. The US Federal Register approved β-CD as GRAS (Generally Recognized as Safe) in 1997 [16, 17].

Many physicochemical methods have been effectively used to characterize the stoichiometric ratio of the inclusion complexes between β-cyclodextrin and a guest, including UV spectroscopy, fluorescence measurements, mass spectrometry, nuclear magnetic resonance (NMR), etc. [18–21]. Electrospray ionization mass spectrometry (ESI–MS) is known for its high accuracy and sensitivity in mass assignment. Therefore, it is particularly suitable to obtain insight into the formation of complex species and their stoichiometry [22–24]. The main aim of our investigation was to examine whether alendronate sodium can form inclusion complexes with β-cyclodextrin, which could possibly be used to improve the bioavailability of alendronate sodium and diminish its side effects. The investigation was performed by way of ESI–MS experiments in order to see whether ions derived from a complex could be formed after introducing a β-CD and sodium alendronate mixture into the mass spectrometer, and by way of molecular modeling.

## Experimental

### Chemicals and reagents

Alendronate sodium was obtained from Cipla Ltd. (India). β-CD was purchased from Cyclolab Ltd. (Hungary) and used without further treatment. All reagents were HPLC grade and were used as such. Acetonitrile and methanol were from Labscan and formic acid (FA) from Park Scientific. Double distilled water was used throughout.

### Mass spectrometry measurements

A complex of alendronate with β-CD was prepared by the addition of an equimolar portion of the sodium alendronate to an aliquot of β-CD in aqueous solution (5 mmol/l). Then the whole solution was shaken for 3 min to mix it effectively before being introduced to the mass spectrometer. All ESI–MS experiments were performed on a MicrOTOF-QII mass spectrometer equipped with an electrospray ion source (Bruker-Daltonics, Bremen, Germany). The following settings were used: electrospray ionization (ESI) in positive and negative ion modes. The dry gas (nitrogen) flow rate was set at 8.0 l/min and the dry heater operated at 180 °C. The capillary voltage was set at 4,500 V and the end plate offset at −500 V. Collision energy was varied in the range of 25–30 eV. MS data were recorded in full scan mode (from 50 to 3,000 *m*/*z*). Data processing was carried out with Chromeleon 6.8. Samples were introduced directly to the electrospray source of the MS using an LC pump and the mobile phase at a flow rate 0.2 ml/min. The mobile phase was composed of H_2_O/ACN/FA (90:10:0.1, v/v/v) (A) and MeOH/ACN/FA (90:10:0.1, v/v/v) (B) in an A:B ratio of 90:10, v/v. HRMS (high resolution) measurements provided by a TOF analyzer to allow for proposing the elemental composition of the registered ions. The assignments of the constituents of the ions are based on the exact masses and isotopic profiles.

### Molecular modeling

Calculations were carried out by Spartan’08 software [25] using the DFT/B3LYP method and the 6-31 + G* basis set. The initial structure of alendronate was optimized with the use of the software. The geometry of β-CD was obtained from its crystallographic structure as determined by X-ray diffraction, which is archived in the Cambridge databank and then optimized at the B3LYP/6-31 + G* level.

Modeling of the complex was performed by docking the optimized structure of the alendronate molecule into the β-CD cavity and allowing for full geometry optimization. Two different inclusion orientations were considered: one with two phosphonate groups bound to the central carbon atom of bisphosphonate inserted into the cavity of βCD from its “top” side (orientation 1) and, another, from its “bottom” side (orientation 2).

∆E of the complex formation was calculated according to Eq. (1):

1 $$ \Updelta {\text{E}} = {\text{E}}_{\text{complex}} {\text{-}} \left( {{\text{E}}_{{\upbeta{\text{-}}{\text{CD}}}}  + {\text{E}}_{{{\text{Aln}}}} } \right) $$

In this equation E_complex_, E_β-CD_ and E_Aln_ are the total energies of the system and of the two separated subsystems, respectively.

## Results and discussion

### Mass spectrometry measurements

**Fig. 2:**
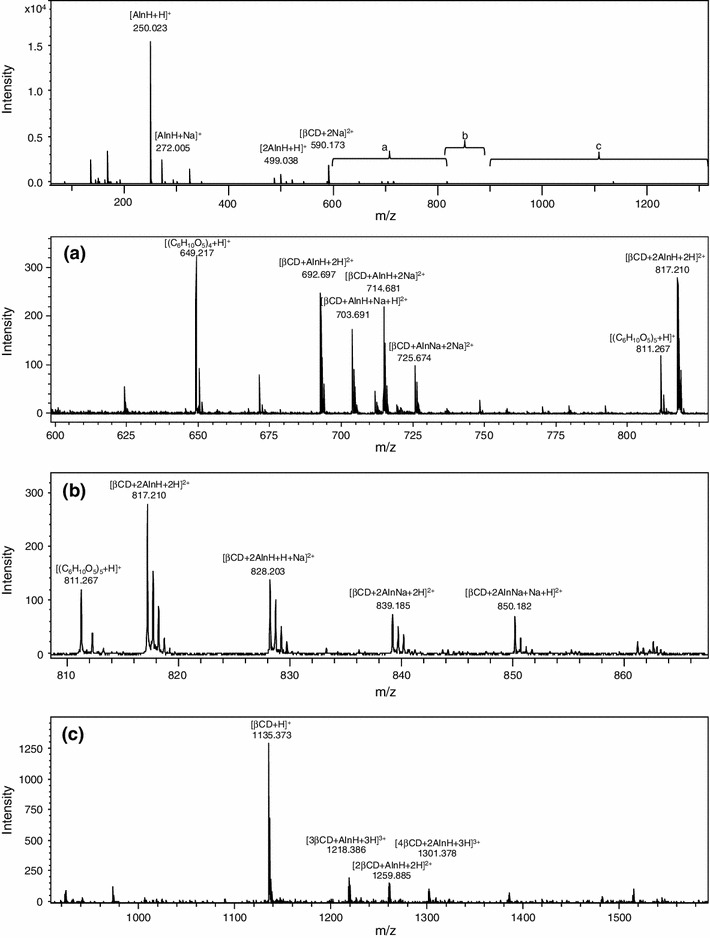
Positive ion ESI mass spectrum for a mixture of β-cyclodextrin and alendronate sodium enhanced in selected ranges

The positive ESI mass spectrum of β-cyclodextrin and alendronate sodium mixture is reported in Fig. 2. The spectrum displays ions formed by complexes of β-cyclodextrin with sodium alendronate and by the pure components of the mixture, sodium alendronate and β-cyclodextrin. One can note two intense peaks of singly charged ions at the *m*/*z* ratios 250.023 and 272.005 corresponding to protonated and sodiated alendronic acid ions: [AlnH + H]^+^ and [AlnH + Na]^+^ and the peak at *m*/*z* 499.038 corresponding to dimeric species of alendronic acid [2AlnH + H]^+^. Doubly charged ions at *m*/*z* 590.173 indicate disodiated β-cyclodextrin ion [βCD + 2Na]^2+^. Small intensity protonated molecular ions of β-cyclodextrin [βCD + H]^+^ were registered at *m*/*z* 1,135.373. The presence of the peak at *m*/*z* ratio 692.697 was attributed to the complex of β-cyclodextrin with alendronic acid [βCD + AlnH + 2H]^2+^ and the peak at *m*/*z* 817.210 to [βCD + 2AlnH + 2H]^2+^, confirming formation of complexes of β-cyclodextrin with alendronic acid. However, those peaks are fairly less intense than the previous ones. The peak at *m*/*z* 692.697 is accompanied by peaks of doubly charged sodiated ions at *m*/*z* 703.691, 714.681 and 725.674. In addition, there are also three less intense peaks present, corresponding to sodiated ions. These are at *m*/*z* 828.203, 839.185 and 850.182, corresponding to [βCD + 2AlnH + H + Na]^2+^, [βCD + 2AlnNa + 2H]^2+^ and [βCD + 2AlnNa + Na + H]^2+^, respectively.

Figure 2c shows many doubly and triple charged ions containing adducts between two and four molecules of β-cyclodextrin and alendronic acid. They result in three small intensity peaks at *m*/*z* 1,259.885, 1,218.386 and 1,301.378, indicating the presence of [2βCD + AlnH + 2H]^2+^, [3βCD + AlnH + 3H]^3+^ and [4βCD + 2AlnH + 3H]^3+^, respectively. Two peaks of singly charged ions at *m*/*z* 649.217 and 811.267 corresponding to [(C_6_H_10_O_5_)_4_ + H]^+^ and [(C_6_H_10_O_5_)_5_ + H]^+^ originate from the fragmentation of β-cyclodextrin.

**Fig. 3:**
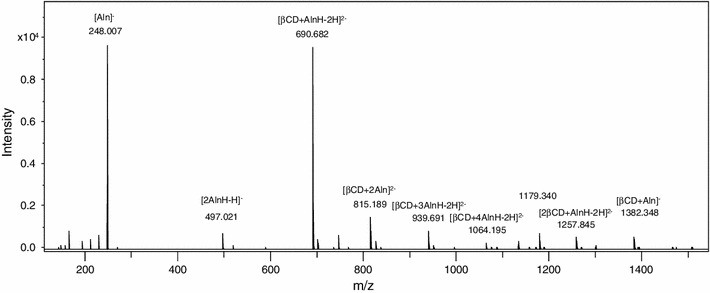
Negative ion ESI mass spectrum for the mixture of β-cyclodextrin and alendronate sodium

The negative ESI mass spectrum of β-cyclodextrin and alendronate sodium mixture is reported in Fig. 3. The main peak in the spectrum at *m*/*z* 248.007 can be attributed to the alendronate anion [Aln]^−^ and the peak at *m*/*z* 497.021 to [2AlnH-H]^−^, the alendronate dimer. Several peaks correspond to the interaction between β-cyclodextrin and alendronate sodium: an intense ion at *m*/*z* 690.682 attributed to [βCD + AlnH-2H]^2−^, *m*/*z* 815.189 to [βCD + 2Aln]^2−^, *m*/*z* 939.691 to [βCD + 3AlnH-2H]^2−^, *m*/*z* 1,064.195 to [βCD + 4AlnH-2H]^2−^, *m*/*z* 1,257.845 to [2βCD + AlnH-2H]^2−^, *m*/*z* 1,382.348 to [βCD + Aln]^−^ and *m*/*z* 1,465.354 corresponding to [3βCD + 4AlnH-3H]^3−^ (very low but with the correct isotopic profile). The singly charged ion at *m*/*z* 1,179.340 was not identified. The total ion current of positive ions was about 20 times higher than that of negative ions.

Tandem mass spectrometry (MS/MS) was used to confirm the structures. The negative ion tandem mass spectrum of the intense doubly charged ion at *m*/*z* 690.682, attributed to [βCD + AlnH-2H]^2−^, shows two peaks corresponding to the alendronate anion [Aln]^−^ at *m*/*z* 248.007 and β-cyclodextrin ion [βCD-H]^−^ at *m*/*z* 1,133.349. MS/MS fragmentation of the protonated [AlnH + H]^+^ alendronate (*m*/*z* 250.023) led to the ion at *m*/*z* 168.041 (elimination of H_3_PO_3_), then *m*/*z* 150.031 (elimination of H_2_O) and *m*/*z* 86.059 (elimination of HPO_2_). MS/MS fragmentation of the alendronate anion [Aln]^−^ (*m*/*z* 248.007) led to the ion at *m*/*z* 148.018 (elimination of H_3_PO_3_ and H_2_O). MS/MS experiments of β-cyclodextrin led to the elimination of subsequent glucose moieties.

These results show that the Aln/β-CD complex is clearly indicated in the negative ion spectrum where it is registered as [βCD + AlnH-2H]^2−^ with a relatively strong peak at *m*/*z* 690.682. Negative ions are more likely to occur because a dissociated anion of the sodium salt is present in the solution. The results indicate not only the 1:1 Aln/β-CD complex, but also complexes with different stoichiometry that had formed in gas phase and could possibly be formed in solution; however, their corresponding peaks were less intense.

### Molecular modeling studies

**Fig. 4:**
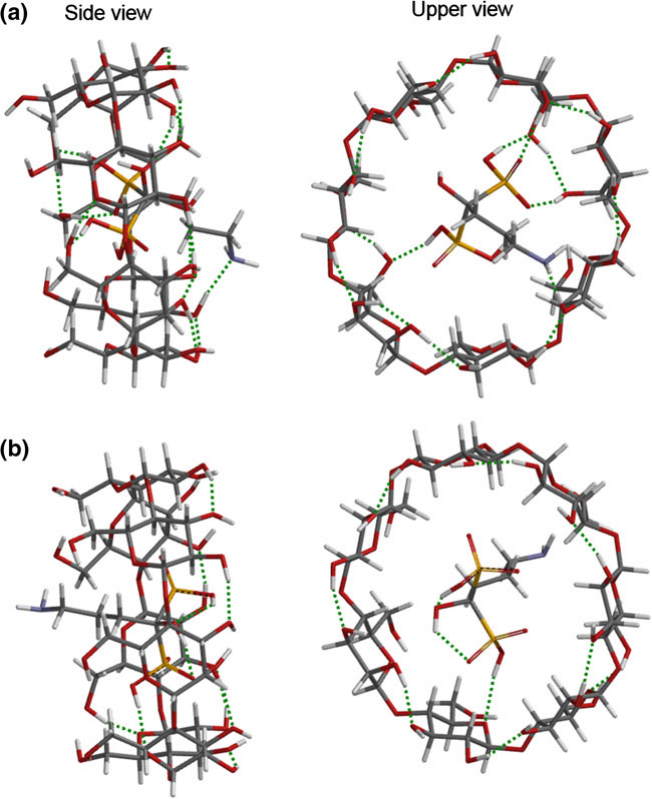
Energy-minimum structures obtained by DFT/B3LYP calculations for the alendronate/β-CD complex in two different orientations: **a** orientation 1, **b** orientation 2. *Dotted lines* represent hydrogen bonds

Calculations using the DFT/B3LYP method were performed in order to obtain some global information about the geometry of the host–guest complexes and to find the intermolecular interaction leading to β-CD and alendronate inclusion complexation. The formed hydrogen bonds, the calculated energies of alendronate and β-CD, the energy of the complex and the complex formation energy (∆E) are shown in Table 1. The ∆E was calculated according to Eq. (1). The optimized structures of the alendronate/β-CD complex of both orientations are shown in Fig. 4.

**Table 1:** The intermolecular hydrogen bonds formed between alendronate and β-CD, their lengths and bond angles, energy of alendronate, energy of β-CD, energies of the Aln-β-CD complexes and energies of the complex formation, ∆E

Orientation	Hydrogen bond (Aln-β-CD)	Bond length (Å)	Bond angle (°)	E_Aln_ (kcal/mol)	E_β-CD_ (kcal/mol)	E_complex_ (kcal/mol)	∆E (kcal/mol)
**1**	P = O···H–O	1.902	158.76	−8,93,503.25	−2,682,715.79	−3,576,293.09	−74.05
	P–O···H–O	1.790	164.11				
	O–H···O	1.699	170.42				
	O–H···O	1.751	159.28				
	N···H–O	1.807	171.37				
**2**	O–H···O	2.073	169.70	−893,503.25	−2,682,715.79	−3,576,279.89	−60.85

After optimization, the alendronate molecule was not completely entrapped in β-CD. The complexation energies were negative, which demonstrates that the inclusion processes of the drug into β-CD were thermodynamically favorable. The lowest values of the complexation energy correspond to the most stable complexes. It is assumed that when the distance between the H atom to a donor (O) and an acceptor (O) is in the range of 0.8–2.8 Å, and the O–H–O angle is above 120.0°, a hydrogen bond is formed [26]. According to this criterion, we noted that the optimized structure in orientation 1 (Fig. 4a) reveals five intermolecular hydrogen bonds formed between the hydrogen atoms of the hydroxyl groups of the drug and the oxygen atoms of the hydroxyl groups of the glucopyranose units of β-CD, between the –OH groups of β-CD and the oxygen atoms of the phosphonate groups, and between the hydroxyl group of β-CD and the nitrogen atom of the drug. The optimized structure with the minimum energy of inclusion complex, i.e. orientation 2 (Fig. 4b), shows that a single intermolecular H bond was formed between a hydrogen atom of the hydroxyl group of the drug and an oxygen atom of the hydroxyl group of β-CD. These results confirm that hydrogen bonding interactions play an important role in the inclusion complexation process.

## Conclusions

This experimental and theoretical study constitutes a physicochemical characterization of the interaction between β-CD and alendronate, a drug from the bisphosphonate group. The specific host–guest complexations between alendronate and β-CD were easily detected by ESI–MS in the gas phase, which suggests that hydrogen bonds were maintained upon vaporization and ionization. Furthermore, molecular modeling showed that the inclusion complex of Aln-β-CD where the two phosphonate groups bound to the central carbon atom of bisphosphonate were inserted into the cavity of β-CD from its “top” side was thermodynamically more favorable than when they were inserted from its “bottom” side. The difference between the two complex formation energies was 13.2 kcal/mol. It can be expected that the present findings will promote extensive future studies into the encapsulation of bisphosphonates to reduce toxicity to the esophageal mucosa.
